# Diagnostic accuracy of the ROCHE Septifast PCR system for the rapid detection of blood pathogens in neonatal sepsis—A prospective clinical trial

**DOI:** 10.1371/journal.pone.0187688

**Published:** 2017-11-08

**Authors:** Julia Straub, Helga Paula, Michaela Mayr, David Kasper, Ojan Assadian, Angelika Berger, Judith Rittenschober-Böhm

**Affiliations:** 1 Department of Pediatrics and Adolescent Medicine, Division of Neonatology, Pediatric Intensive Care and Neuropediatrics, Medical University Vienna, Vienna, Austria; 2 Department of Hospital Epidemiology and Infection Control, Medical University Vienna, Vienna, Austria; 3 Department of Pediatrics and Adolescent Medicine, Research Core Unit of Pediatric Biochemistry and Analytics, Medical University Vienna, Vienna, Austria; University of Illinois at Urbana-Champaign, UNITED STATES

## Abstract

**Introduction:**

Diagnosis of neonatal sepsis remains a major challenge in neonatology. Most molecular-based methods are not customized for neonatal requirements. The aim of the present study was to assess the diagnostic accuracy of a modified multiplex PCR protocol for the detection of neonatal sepsis using small blood volumes.

**Methods:**

212 episodes of suspected neonatal late onset sepsis were analyzed prospectively using the Roche SeptiFast^®^ MGRADE PCR with a modified DNA extraction protocol and software-handling tool. Results were compared to blood culture, laboratory biomarkers and clinical signs of sepsis.

**Results:**

Of 212 episodes, 85 (40.1%) were categorized as “not infected”. Among these episodes, 1 was false positive by blood culture (1.2%) and 23 were false positive by PCR (27.1%). Of 51 (24.1%) episodes diagnosed as “culture proven sepsis”, the same pathogen was detected by blood culture and PCR in 39 episodes (76.5%). In 8 episodes, more pathogens were detected by PCR compared to blood culture, and in 4 episodes the pathogen detected by blood culture was not found by PCR. One of these episodes was caused by *Bacillus cereus*, a pathogen not included in the PCR panel. In 76/212 (35.8%) episodes, clinical sepsis was diagnosed. Among these, PCR yielded positive results in 39.5% of episodes (30/76 episodes). For culture-positive sepsis, PCR showed a sensitivity of 90.2% (95%CI 86.2–94.2%) and a specificity of 72.9% (95%CI 67.0–79.0%).

**Conclusion:**

The Roche SeptiFast^®^ MGRADE PCR using a modified DNA extraction protocol showed acceptable results for rapid detection of neonatal sepsis in addition to conventional blood culture. The benefit of rapid pathogen detection has to be balanced against the considerable risk of contamination, loss of information on antibiotic sensitivity pattern and increased costs.

## Introduction

Neonatal sepsis is still one of the leading causes of neonatal mortality; yet, confirming the clinical suspicion of neonatal sepsis remains challenging [1, 2]. Up to 20% of very low birth weight infants (VLBWI) may acquire at least one episode of culture-positive sepsis during the hospital stay [3], with an incidence of up to 50% for clinical sepsis without microbiological confirmation [3–8]. Alongside the high mortality depending on the causative microorganism [9, 10], other complications include broncho-pulmonary dysplasia, neurodevelopmental impairment, prolonged hospital stay and increased risk for re-hospitalization [3, 4, 6, 9–13]. Several studies have demonstrated the beneficial impact of early and adequate treatment of neonatal sepsis in order to reduce mortality of preterm infants [11–13]. The basis for early treatment is confirmation of the clinical suspicion of neonatal sepsis together with, if possible, identification of the causative microorganism.

However, laboratory and clinical signs of neonatal sepsis are typically uncharacteristic [14, 15], and hence, empiric antibiotic treatment is initiated frequently [16, 17]. This again may have a negative impact on the likelihood of positive microbial culture and microbiological detection of the causative agent.

Although a positive blood culture and conventional bacteriologic methods still are the “gold standard” to ascertain the clinical suspicion of neonatal sepsis, this approach is limited by the time of the availability of results and, particularly in neonates, the low sensitivity due to the very small sampling volumes obtainable [18, 19]. These limitations may be overcome with molecular PCR based rapid diagnostic tests for bacterial detection [20].

Although modern PCR techniques provide rapid test results within 5–6 hours, resulting in targeted pathogen identification and decreased use of broad-spectrum antibiotics [21, 22], such technology derives from adult intensive care where the obtainable volume of samples is larger [23, 24]. Consequently, molecular based detection methods have also been introduced as an additional tool for the diagnosis of neonatal sepsis, yet, blood volumes were not adapted to neonatal requirements [25–26], and required sample volumes still range form 1 mL to 3 mL of blood.

Recently, we have reported on a modification DNA extraction protocol for the ROCHE multiplex PCR to detect blood stream pathogens in neonatal sepsis [27]. This modified DNA extraction technique yielded a higher sensitivity (90.5%) but lower specificity (80.0%) for rapid molecular detection of bacterial pathogens in blood volumes as low as 100 μL as compared to 1.5 mL to 3 mL of blood for conventional blood culture [27]. The feasibility of such DNA extraction modification protocol was supported recently [28].

The aim of the present prospective clinical trial was to assess the clinical feasibility and practicability of the ROCHE SeptiFast MGRADE system used with a modified DNA extraction protocol for diagnosis of sepsis in preterm infants in addition to conventional blood culture.

## Material and methods

From June 2010 to May 2012, all neonates admitted to the neonatal intensive care units of the Department of Neonatology, Pediatric Intensive Care and Neuro-pediatrics of the Medical University of Vienna, who survived beyond 72 hours of life were encompassed into this study. The ethic committee of the Medical University of Vienna approved the study (EK Votum No.: 428/2010) and written informed consent of the parents was obtained.

### Clinical definitions

Clinical characteristics of patients and clinical symptoms of infection were extracted from electronic medical records. Patients’ baseline data and demographics are summarized in Table 1. Neonatal sepsis episodes were compared with blood culture results and clinical as well as laboratory parameters of infection. Clinical sepsis was defined following the NEO-KISS surveillance definitions [8, 16]. Individual patient cases were allocated to one of the following three study groups:

Group 1 (control, no sepsis):

- No laboratory signs of sepsis, negative blood culture.
- Antibiotics, if started, discontinued after a maximum of three days.

Group 2 (blood culture proven sepsis):

- Sepsis with clinical signs of infection PLUS
- Positive blood culture result PLUS
- CRP > 2 mg/L or I:T neutrophil ratio > 0.2 in case of coagulase negative streptococci (ConS) sepsis

**Table 1:** Baseline characteristics of study population.

N	Total	Sepsis not suspected	Sepsis suspected	P-value
	206	89	117	
Gestational age (d ± SE)	198.9 (± 2.6)	205.7 (± 2.5)	193.7 (± 1.9)	0.001
Birth weight (g ± SE)	1,161.3 (± 0.07)	1,251.2 (± 0.05)	1,092.9 (± 0.05)	0.088
APGAR 1 min (± SE)	8.2 (± 0.08)	8.3 (± 0.09)	8.1 (± 0.12)	0.243
Female N (%)	96 (46.6)	43 (48.3)	53 (45.3)	0.485
Single birth N (%)	126 (61.2)	39 (43.8)	87 (74.4)	< 0.001
Antenatal steroids No—N (%)	29 (14.1)	10 (11.2)	19 (16.2)	0.651
Antenatal steroids Incomplete—N (%)	70 (34.0)	37 (41.6)	33 (28.2)	0.076
Antenatal steroids Completed—N (%)	107 (51.9)	42 (47.2)	65 (55.6)	0.261
Maternal infection N (%)	85 (41.3)	28 (31.5)	57 (48.7)	0.009

SE = Standard error of means

Additionally, ConS, *Corynebacterium species*, alpha- or non-hemolytic streptococci, *Bacillus species*, *Propionibacterium acnes*, micrococci, and *Neisseria species* other than *N*. *gonorrhoeae* and *N*. *meningitidis* were defined as possible skin contaminants. Other bacteria and fungi were regarded as obligate pathogens (e.g. *Salmonella typhi*, *Staphylococcus aureus*) and therefore were always considered as true cause of bacteremia, whereby only the first isolate within a 14-day period was counted as one episode.

Bacteremia caused by a possible skin contaminant organism was assumed as true if an organism of the same species with a similar antimicrobial susceptibility pattern (at least 85% similarity) was isolated from two or more sets of blood cultures obtained from the same patient within 5 days starting from the last positive culture with the identical organism. In this case, this was counted as a single episode of bacteremia.

Group 3 (clinical sepsis):

- at least 2 clinical signs of sepsis PLUS
- at least one positive laboratory parameter for infection (CRP > 2 mg/L, I:T neutrophil ratio > 0.2, IL-8 > 100 pg/μL) OR CRP > 1.5 mg/mL AND I:T neutrophil ratio > 0.15 PLUS
- Anti-infective therapy for at least 5 days PLUS
- No other focus of infection PLUS
- Negative blood culture.

### Neonatal sepsis-workup

If neonatal sepsis was suspected based on clinical signs and symptoms, routine sepsis workup included sampling of blood culture as well as hematology and clinical chemistry. Due to the limited amount of blood available, only the aerobic pediatric blood culture bottle (BacT/Alert^®^ blood culture bottles; BioMerieux, Marcy l'Etoile, France) was inoculated. Blood was taken through peripheral puncture of the vein after skin disinfection with an alcohol based skin antiseptic. 0.5 to 1 mL of peripheral blood was used for blood culture. Blood cultures were incubated for at least 7 days. Positive blood cultures were Gram stained and detected microorganisms were further identified to the species level following standard microbiological methods [29]. In addition, 200 μL EDTA blood was used for automated hematological analysis (XE2100, Sysmex, Kobe, Japan) and manual cell differentiation of a full blood smear was performed. 100 μL of serum were taken for clinical chemistry, including determination of CRP and Interleukin 8 (IL-8—VITROS^®^ 5,1 FS Chemistry System, Ortho-Clinical Diagnostics, N.J., USA).

### PCR processing

In study patients, an additional blood sample of at least 100 μL of EDTA blood was collected into a 0.8 mL K3 EDTA sampling tube (Sarstedt Vacutainer GmbH, Nümbrecht, Germany) for PCR analysis. Samples were processed at the Research Core Unit of Pediatric Biochemistry and Analytics, Medical University Vienna. The Roche LightCycler^®^ SeptiFast MGRADE is a commercially available certified multiplex real-time PCR system with simultaneous analysis of 20 different pathogens (Table 2) which are commonly retrieved pathogens in neonatal units [19, 22]. PCR analysis was processed from 100 μL EDTA blood using the LightCycler^®^ SeptiFast MGRADE system (Roche Diagnostics, Penzberg, Germany) with a modified DNA extraction protocol as previously described [27]. Briefly, the three principal steps are: *i*) DNA extraction and purification, modified for the use of small blood volumes: 100 μL blood samples (minimal volume determined in preliminary study) were enhanced to a volume of 1.5 mL (as used in the manufacturer's standard protocol [30]) with a 1:1 mixture of nucleic acid free water (Fermentas, Burlington, Canada) and Roche Negative Control in the K3EDTA blood collection tube and processed in an sterile environment. *ii*) PCR processing for bacteria (Gram-positive and Gram-negative) and fungi in three parallel reactions; *iii*) Detection of amplified PCR products via Internal Transcibed Spacer (ITS) region (bacterial ribosomal RNA: between 16S and 23S, fungal ribosomal RNA: between 18S and 5.6S [30]) by hybridization probes and melting temperature (Tm) curve analysis.

**Table 2:** SeptiFast MGRADE system pathogen spectrum.

Gram-positive	Gram-negative	Fungi
*Staphylococcus aureus*	*Escherichia coli*	*Aspergillus fumigatus*
Coagulase negative Staphylococci ^1^	*Klebsiella pneumoniae/oxytoca*	*Candida albicans*
*Streptococcus pneumoniae*	*Serratia marcescens*	*Candida glabrata*
*Streptococcus spp*. ^2^	*Enterobacter cloacae/aerogenes*	*Candida krusei*
*Enterococcus faecalis*	*Proteus mirabilis*	*Candida parapsilosis*
*Enterococcus faecium*	*Pseudomonas aeruginosa*	*Candida tropicalis*
	*Acinetobacter baumannii*	
	*Stenotrophomonas maltophilia*	

^**1**^ *S*. *epidermidis*, *S*. *haemolyticus*, *S*. *hominis*, *S*. *pasteuri*, *S*. *warneri*, *S*. *cohnii*, *S*. *lugdunensis*, *S*. *capitis*, *S*. *caprae*, *S*. *saprophyticus*, *and S*. *xylosus*.

^**2**^ *S*. *agalactiae*, *S*. *pyogenes*, *S*. *anginosus*, *S*. *bovis*, *S*. *constellatus*, *S*. *cristatus*, *S*. *gordonii*, *S*. *intermedius*, *S*. *milleri* group, *S*. *mitis*, *S*. *mutans*, *S*. *oralis*, *S*. *parasanguinis*, *S*. *salivarius*, *S*. *sanguinis*, *S*. *thermophilus*, *S*. *vestibularis*, S. viridans group

### Automated work-up of PCR results

For analysis of PCR signals, the SeptiFast Identification Software (SIS; Roche Diagnostics, Penzberg, Germany), an automatic PCR analysis system designed for blood volumes of at least 1 mL, based on cycle threshold numbers (CTs), was used. CT values were inversed to the number of nucleoid acids measured and correlate to the number of copies. For the exclusion of potential (cross-) contaminations with skin bacteria such as ConS and *Streptococcus spp*., none of the integrated algorithms recognizes CT values for these pathogens above 20. To re-evaluate SIS [27], ConS-infections were analyzed and compared to manual CT analyses results.

### Statistics

For statistical analyses of analytic and demographic variables Excel for Macintosh 2011 (Microsoft Inc., Redmond, WA) and SPSS 21.0 (SPSS Science, Wash., USA) were used. Continuous variables were described as mean ± standard deviation (SD). The p-value was set at p < 0.05, indicating statistical significance.

## Results

206 patients were included in the study after written consent of the parents was obtained. Screening for sepsis was performed in 117 of these 206 patients. Among the remaining 89 patients, 61 patients did not present any sign of sepsis during their stay at the neonatal intensive care unit, and in 28 patients blood culture sampling was done without taking additional blood for PCR sampling. This situation typically occurred during night shifts. Forty-five oft the 117 patients presented with more than 1 episode of clinically suspected sepsis, resulting in 212 episodes for final analysis.

### Group 1 (no sepsis, n = 85)

23/85 episodes screened for sepsis had a positive PCR result (ConS: n = 22, ConS and *Escherichia coli*: n = 1). One infant had a contaminated blood culture (ConS/ *Staphylococcus capitis*). In this case, clinical and laboratory signs of sepsis were missing, and antibiotic treatment was discontinued within three days.

### Group 2 (blood-culture positive sepsis, n = 51)

46/ 51 pathogens detected in conventional blood culture were correctly detected by PCR. In 8/46 episodes, more than one pathogen was detected through PCR (2 pathogens in five episodes of sepsis, 3 pathogens in two episodes, and 4 pathogens in one episode). 5/51 pathogens detected through conventional microbiology were missed by PCR: One patient presented with sepsis caused by *Bacillus cereus*, which is not included in the SeptiFast panel. In 4 further episodes PCR also remained false negative (Table 3).

**Table 3:** Positive blood cultures missed by SeptiFast MGRADE system.

Patient ID	Result Blood Culture	Result PCR Gr+	Result PCR Gr-	Result PCR Fungi
# 31	*S*. *epidermidis*	Negative	Negative	Negative
# 85	*S*. *capitis*	Negative	Negative	Negative
# 105	*S*. *aureus*	ConS	Negative	Negative
# 129	*B*. *cereus*	Negative	Negative	Negative
# 143	*E*. *coli*	Negative	Negative	Negative

### Group 3 (clinical sepsis, n = 76)

30/76 (39.5%) neonates with clinical sepsis had a positive PCR result (ConS: n = 24, *Streptococcus sp*.: n = 2). The two episodes with *Streptococcus sp*. had a maximum CRP at 4.2 and 4.6 mg/dL, respectively. A polymicrobial sepsis with ConS, *E*. *faecalis*, *K*. *oxytoca* and *E*. *cloacae* was detected in one episode with a maximum CRP at 10.5 mg/dL. A further episode presented with *K*. *pneumonia* and *E*. *coli* with a CRP of 19.5 mg/dL; two more episodes of clinical sepsis without yield of a causative agent in conventional blood culture but positive PCR signal were caused by *E*. *faecalis* (CRP = 3.8 mg/dL) and *S*. *aureus* (CRP = 31.2 mg/dL).

In 145 /212 (67%) of all episodes, PCR and blood culture produced same results (group 1—control, no sepsis: 61 episodes with congruent negative PCR and blood culture; group 2—blood culture proven sepsis: 38 episodes with same pathogen in PCR and blood culture; and group 3—clinical sepsis: 46 episodes with negative PCR and blood culture results).

Defining a clinically ill patient as gold standard (clinical sepsis together with blood-culture positive sepsis), PCR performed with a modified DNA extraction protocol showed a sensitivity of 60% (95% CI = 53%–66%) and a specificity of 73% (95% CI = 67%–79%) compared to a sensitivity of 40% (95% CI = 34%–47%) and a specificity of 99% (95% CI = 97%–100%) in blood cultures.

Regarding the diagnostic accuracy of clinical parameters and a positive blood culture verifying the clinical suspicion of sepsis, only group 1 (no sepsis) and group 2 (blood culture proven sepsis) were compared in a further step. Clinical suspicion combined with positive blood culture increased the sensitivity of the PCR system to 90% (95% CI = 86%–94%, Table 4).

**Table 4:** Comparison of SeptiFast MGRADE PCR and blood culture results.

PCR results in patients without sepsis vs. culture proven sepsis: Sensitivity = 0.9020; Specificity = 0.7294			
	No sepsis	Blood culture proven sepsis	Total
FP	23	-	23
TP	-	46	46
FN	-	5	5
TN	62	-	62
Total	85	51	136

However, in patients with clinical signs of sepsis (clinical sepsis and *blood-culture positive sepsis*), 60% of blood culture (n = 76) resulted in negative PCR tests, whereas only 40% of PCR results (n = 51) were blood culture negative. This finding may be explained by the higher detection rate for Gram-positive pathogens in the PCR panel (59%, n = 75 vs. 39%, n = 49), and was mainly contributed by detection of ConS (55%, n = 70 vs. 31%, n = 39). For blood culture proven sepsis (group 2), the detailed results for conventional microbiology and PCR are depicted in Table 5. PCR was particularly able to identify ConS, *S*. *aureus* and *E*. *coli* sepsis.

**Table 5:** Detection of ConS, *S*. *aureus* and *E*. *coli*, stratified by blood culture and PCR.

	Blood culture N (%^2^)		PCR N (%^2^)	
ConS				
Total ConS	41	32.3	69	54.3
False positive^3^	1	0.8	23	18.1
ConS only	40	31.5	60	47.2
Multiple isolates^1^	1	0.8	9	7.1
*S*. *aureus*				
Total *S*. *aureus*	5	3.9	7	5.5
*S*. *aureus* only	4	3.2	1	0.8
Multiple isolates^1^	1	0.8	6	4.7
*E*. *coli*				
Total *E*. *coli*	2	1.6	3	2.4
False positive^3^	0	0.0	1	0.8
*E*. *coli* only	1	0.8	1	0.8
Multiple isolates^1^	1	0.8	2	1.6

^1^ pathogen detected as one of multiple isolates

^2^% of isolations in blood culture proven or clinical sepsis episodes (n = 127)

^3^ false positive results are calculated based on total episodes

### Analysis of CONS-infections compared to automatic SIS—analysis

For patients with no sepsis (group 1) but positive PCR results with typical skin contaminants, manual analyzed CT-values for ConS ranged between 16 and 27 with two values below 20, which were also detected by SIS (automatic CT-Value 17 and 19, respectively). Twenty samples positive for ConS in group 3 (patients with clinical sepsis but negative blood cultures) showed manual CT-values between 18 and 26, with 2 samples below 20, which were also detected by SIS (manual CT-Value 18 and 20, respectively). The range of CT levels differed between 13 and 26 out of 45 samples (manual CT-Values all < 20) and were correctly detected by SIS.

## Discussion

Despite a progress in sepsis treatment and reduction of sepsis rates in neonatology in the past years [1, 10], late-onset sepsis remains one of the major challenges and a common problem in neonatal intensive care units. Due to the immaturity of the immune system, incidence of sepsis and infections in neonates range up to 50% [3–8], and is associated with a higher risk for mortality, severe long-term morbidities and poor neurodevelopmental outcome [3, 4, 6, 9–13]. To overcome these risks, calculated and broad antimicrobial treatment is initiated rapidly. However, prolonged and broad-spectrum antibiotic treatment not only results in impaired neonatal outcome with higher individual risk for comorbidities, fungal sepsis and necrotizing entero-colitis, but also entails the global risk of increasing antibiotic resistance [14, 15]. Therefore, diagnosis of neonatal sepsis should offer results promptly and as accurately as possible in order to administer subsequent antibiotic treatment rationally, suitably and timed.

Although there is lack of consent in a specific definition of neonatal sepsis [1], current best practice requires either the same pathogen isolated from two blood cultures or elevated laboratory signs of infection combined with a positive blood culture [6]. This state-of-the-art procedure particularly applies for the diagnosis of ConS sepsis. Nonetheless, due to small blood volumes and difficulties in sampling in neonatal patients, a routine sepsis workup in preterm infants commonly comprises only one blood culture. Therefore, and due to small blood volumes, suppression of bacterial growth by preceding antibiotic treatment and low-concentration bacteremia, culture samples of clinically septic patients turn out to be negative, which then commonly is referred to as “clinical sepsis” [1, 2, 27, 28].

Results of our present study show that the commercially available SeptiFast MGRADE PCR system can be implemented in clinical neonatology using a modified sample handling and DNA extraction protocol with blood samples up to 100 μL of volume, which might increase the detection rate of pathogens in patients with “clinical sepsis”. Furthermore, prompt result availability within 6 hours is a crucial advantage of the Septifast system in critically ill and instable patients [27, 28, 31–33]. In our study, 40.2% of the 127 blood cultures obtained from patients retrospectively considered as ill turned out to be positive, whereas the ratio of positive PCR results (59.8%) was higher. Both findings—a higher ratio of positive PCRs and positive blood culture results in less than half of all ill patients—are similar to other studies comparing PCR techniques with blood cultures [19, 21, 23–28, 31–34]. In 66.5% of all episodes, PCR and blood culture provided the same—either positive or negative—result, which is comparable to other studies with concordances reported between less than 60% and > 75% [23, 28, 33, 34].

Our study has a number of limitations. There were a considerable number of episodes with false positive PCR results. This circumstance can be attributed to the high likelihood of DNA-contamination during blood sampling in neonates and sample processing mainly with skin bacteria, a common problem of PCR techniques due to high PCR sensitivity and likewise found in other PCR studies which makes adequate blood sampling a major challenge [19, 21–23, 25, 28, 31, 33–35]. This particularly applies for detection of ConS, as it was the dominating pathogen causing false positive PCR results (ConS: 22/85, ConS and *E*. *coli*: 1/85). However, the problem of contamination of blood samples pertains also for non-PCR-based detection methods, such as conventional blood cultures.

Five episodes of culture-positive sepsis were not detected by PCR. Although one of these episodes was caused by *B*. *cereus*, a pathogen not included in the SeptiFast PCR panel, 4 other blood pathogens included in the SeptiFast panel were equally not detected. A similar finding was observed by others [28], who reported even a trend-wise higher ratio of false negative PCR results as compared to our study. Tröger et al. [28] explained such false negative PCR findings with possible “genetic variability among the pathogens, or mutations of the target site, inappropriate sample preparation or prolonged transport times” [28].

Our study did not consider whether a patient historically was exposed to antibiotic therapy (for prophylactic reasons after birth or due to a previous sepsis episode) before the sepsis episode blood was taken for study proposes. Hence, no statement can be made on the likelihood of negative blood culture and / or PCR in cases of clinical infection or of the probability of false negative PCRs in cases of culture proven sepsis. Due to the fact that in our neonatal department all patients born less than 28 weeks of gestation receive prophylactic therapy with ampicillin and gentamicin for at least 48 hours, a previous exposure to antibiotics is very likely.

Although the SeptiFast PCR system includes fungal pathogens frequently encountered in neonatal intensive care units, the potential advantage of PCR based diagnostics could not be evaluated since no episodes of fungal sepsis occurred in our study. This is most likely caused by the practice of fluconazole prophylaxis in all preterm infants up to 27 weeks of gestational age at our neonatal intensive care unit, which represent the group with the highest risk for fungal sepsis among our neonatal units.

As the volume of blood taken for cultures and the amount of cultures correlate with their sensitivity, another reason for a potential limitation in sensitivity of blood culture results could be the fact that blood samples were neither standardized nor was their volume measured. However, at our units clinicians admittedly have the advice to sample at least 0.5 mL for blood culture.

Our previous suspicion [27] of unreliability of automated identification software SIS (designed and certified for blood volumes of at least 1 mL) was confirmed: Although SIS provides a potential improvement against false identification of contamination due to the lower sensitivity caused by higher PCR detection limits of skin bacteria, lower blood volumes resulted in higher CT values, which exceeds threshold basis of this automated SIS-calculation. Therefore, with the adapted protocol, SIS is not reliable in pathogen detection and manual analysis conducted by an experienced staff well trained in PCR analysis has to be done.

A general limitation of all molecular-based detection methods is that they do not offer specification of pathogens, therefore no further testing in anti-infective sensitivity and resistance and no pathogen-targeted therapy can be implemented with pathogen detection solely from PCR sampling. As these very sensitive PCR methods cannot discriminate the origin of the DNA measured (living vs. dead bacteria, contamination vs. infection), higher rates of false positive results are created. Therefore, and what was highlighted elsewhere [34], PCR results always have to be interpreted in the clinical context.

A further limitation is the fact that multiplex PCR tests still remain a cost- and staff- intense procedure, as PCR costs are 5-times higher compared to conventional blood cultures costs, and staff needs special cost- and time-intense training.

Considering these limitations, the PCR system cannot be recommended as replacement of conventional microbiology but as a powerful additional tool, increasing the overall sensitivity of sepsis diagnosis. Nevertheless, PCR provides a potential benefit concerning one limitation of blood culture as a gold standard in sepsis diagnosis: After initiation of anti-infective therapy, bacterial blood cultures often remain negative; here PCR techniques could offer important additional information on possibly present, but non-culturable pathogens [31].

## Conclusion

Our study demonstrates that, in comparison to routinely used blood culture techniques, the Roche SeptiFast MGRADE multiplex PCR system applying a modified DNA extraction protocol with a decreased blood volume of 100 μL provides:

1. 90.2% sensitivity for the diagnosis of blood-culture positive sepsis episodes,
2. a moderately high number of positive test results in episodes without infection potentially due to contamination during blood drawing and/or processing, and
3. an increased pathogen detection rate in patients with clinical sepsis.
